# Nursing Skill Mix, Nurse Staffing Level, and Physical Restraint Use in US Hospitals: a Longitudinal Study

**DOI:** 10.1007/s11606-016-3830-z

**Published:** 2016-08-23

**Authors:** Vincent S. Staggs, Danielle M. Olds, Emily Cramer, Ronald I. Shorr

**Affiliations:** 1 Health Services and Outcomes Research, Children’s Mercy Hospitals and Clinics, 2401 Gillham Rd, Kansas City, 64108 MO USA; 2 School of Medicine, University of Missouri-Kansas City, 2411 Holmes St, Kansas City, MO 64108 USA; 3 School of Nursing, University of Kansas Medical Center, 3901 Rainbow Blvd, Kansas City, KS 66160 USA; 4 Geriatric Research, Education and Clinical Center (GRECC), Malcom Randall Veterans Administration Medical Center, Gainesville, FL USA; 5 Department of Epidemiology, University of Florida, 2004 Mowry Road, PO Box 100231, Gainesville, FL 32610 USA

**Keywords:** physical restraint, nurse staffing, patient safety, falls, quality of care

## Abstract

**Background:**

Although it is plausible that nurse staffing is associated with use of physical restraints in hospitals, this has not been well established. This may be due to limitations in previous cross-sectional analyses lacking adequate control for unmeasured differences in patient-level variables among nursing units.

**Objective:**

To conduct a longitudinal study, with units serving as their own control, examining whether nurse staffing relative to a unit’s long-term average is associated with restraint use.

**Design:**

We analyzed 17 quarters of longitudinal data using mixed logistic regression, modeling quarterly odds of unit restraint use as a function of quarterly staffing relative to the unit’s average staffing across study quarters.

**Subjects:**

3101 medical, surgical, and medical-surgical units in US hospitals participating in the National Database of Nursing Quality Indicators during 2006–2010. Units had to report at least one quarter with restraint use and one quarter without.

**Main Measures:**

We studied two nurse staffing variables: staffing level (total nursing hours per patient day) and nursing skill mix (proportion of nursing hours provided by RNs). Outcomes were any use of restraint, regardless of reason, and use of restraint for fall prevention.

**Key Results:**

Nursing skill mix was inversely correlated with restraint use for fall prevention and for any reason. Compared to average quarters, odds of fall prevention restraint and of any restraint were respectively 16 % (95 % CI: 3–29 %) and 18 % (95 % CI: 8–29 %) higher for quarters with very low skill mix.

**Conclusions:**

In this longitudinal study there was a strong negative correlation between nursing skill mix and physical restraint use. Ensuring that skill mix is consistently adequate should reduce use of restraint.

## INTRODUCTION

Physical restraint is a common, undesirable occurrence in health care.^1^^–^ 4 Defined by the Centers for Medicare and Medicaid Services (CMS) as “any manual method, physical or mechanical device, material, or equipment attached to or adjacent to the resident’s body that the individual cannot remove easily which restricts freedom of movement or normal access to one’s body,”^5^ physical restraints can include belts, mittens, vests, bedrails, geriatric chairs, and other devices. Use of such devices has come under intense scrutiny, as physical restraint can result in agitation, confusion, deconditioning, pressure ulcers, strangulation, death, and adverse psychological effects.^3^ Due to these serious consequences, physical restraint use is part of public reporting for nursing homes through the CMS Nursing Home Compare website,^6^ and researchers have begun to examine correlates of restraint use in various settings.^1^^,^ 2 ^,^ 7

Nurse staffing is a focus of patient outcomes research because of nurses’ role in providing direct care to patients. Nurse staffing variables have been linked to a number of patient outcomes and to patient satisfaction.^8^^–^ 17 Researchers have studied measures of both quantity and quality of staffing, including nurse-to-patient ratios, nursing hours per patient day, and nursing skill mix, typically defined as the proportion of total nursing hours worked by RNs.^18^^–^ 20

Most studies examining nurse staffing as a correlate of restraint use have focused on nursing homes.^21^^–^ 27 The relation between staffing and restraint use in hospitals is not well studied, and results of research to date are inconclusive, perhaps because of inadequate control for unobserved differences among units (e.g., patient mix).^28^^–^ 30 Our aim was to examine associations of nurse staffing level and skill mix with use of restraints.

## METHODS

### Sample and Data

The National Database of Nursing Quality Indicators (NDNQI) provided quarterly data on restraints and monthly data on nurse staffing from medical, surgical, and medical-surgical units in US acute care hospitals for the 17-quarter study period October 2006–December 2010. NDNQI participation is voluntary. Data were collected with oversight from the Human Subjects Committee at the University of Kansas Medical Center.

NDNQI hospitals report nurse staffing data monthly for participating units. These data include the monthly numbers of nursing care hours worked (not scheduled) by RNs, licensed practical/vocational nurses, and assistive personnel assigned to the unit. Hours worked by students and by nursing personnel who do not spend >50 % of their time in direct care are excluded. Hospitals also report the monthly patient census for each unit.

NDNQI hospitals collect data on physical restraints during a survey carried out quarterly on each participating unit. On the designated survey day, unit personnel record the total number of patients surveyed, the age and sex of each patient surveyed, and the count of patients restrained. For each restrained patient, unit personnel indicate the clinical justification for restraint by choosing one or more of the following: prevent from falling by getting out of bed without assistance, prevent from removing medical equipment or therapeutic modalities, reduce potential for inflicting harm to others, reduce potential for inflicting harm to self, other, and unknown. These data, along with the unit census on the day of the survey, are reported to the NDNQI.

Of 6279 units in the original data set reporting restraint data during the study period, 5503 reported both restraint and staffing data for at least two quarters. To allow us to explore within-unit differences in staffing between quarters with and without reported use of restraints with each unit serving as its own control, the sample was further limited to units with at least one quarter in which restraint use was reported for one or more patients and at least one quarter in which restraint use was not reported for any patient. Data from units excluded for reporting no restraint use in any quarter or for using restraint in every quarter were retained for comparison to the study sample, which comprised 3101 units from 869 hospitals.

### Variables

#### Outcomes

We examined two dichotomous outcomes in the study: one indicator of whether restraint use for any purpose was reported for at least one patient on the unit in the quarterly survey, and one indicator of whether restraint use for the purpose of fall prevention was reported. For descriptive purposes we also computed each unit’s overall restraint prevalence (proportion of total patients assessed during the study who were restrained) and fall prevention restraint prevalence (proportion of patients assessed who were restrained for fall prevention).

#### Nurse Staffing Variables

As a measure of staffing level we computed quarterly total nursing hours per patient day (TNHPPD) for each unit as the sum of total nursing care hours for the quarter divided by total inpatient days for the quarter. In addition to computing each unit’s mean staffing level across quarters, we categorized each unit’s quarterly staffing level based on its distance in standard deviations (SDs) above or below this mean. Quarters were then classified as “average” for levels within 0.5 SDs of the mean, “low” (“high”) for levels 0.5–1.25 SDs below (above) the mean, and “very low” (“very high”) for levels >1.25 SDs below (above) the mean.

We computed each unit’s quarterly skill mix as the proportion of quarterly total nursing care hours provided by RNs. As with TNHPPD, these values were standardized for each unit to reflect the difference in SDs between the quarterly skill mix and the unit’s mean skill mix across study quarters and used to classify each quarter as “very low,” “low,” etc.

#### Covariates

Hospital demographic variables included US census region, bed size (<300 or ≥300 beds), teaching status, and location (metropolitan or non-metropolitan). Two indicator variables for unit type were used to compare odds of restraint for medical, surgical, and medical-surgical units. To provide some control for within-unit differences in patient mix, we computed the mean age of patients assessed and the proportion of patients assessed who were male from the quarterly survey for each unit quarter. Mean age and proportion male were coded as missing for quarters in which the relevant information was available for fewer than 25 % of patients assessed. Patient volume was measured as quarterly patient days.

### Analyses

#### Restraint for Any Reason

In the first analysis we analyzed data for the full study sample (units having at least one quarter with and one quarter without restraint use). Using mixed logistic regression models fit with the GLIMMIX Procedure in SAS 9.4, we estimated the odds of a unit reporting restraint use in a quarter as a function of staffing level and skill mix. Random unit and hospital intercepts were included to account for correlation due to clustering. Explanatory variables included hospital characteristics (census region, bed size, teaching status, and location), patient volume, mean staffing level (TNHPPD), staffing level category (very low, low, etc.), mean skill mix, skill mix category, unit type, average age, and proportion male. Year and quarter were included as classification variables to control for time trend and seasonality.

Staffing level category and skill mix category were treated as classification variables to allow for easily interpretable non-monotonic associations between staffing and odds of restraint. For both variables we set “average” as the referent category and estimated odds ratios for the other four categories. Because these classification variables were based on standardized values of TNHPPD and skill mix, they reflected only within- (not between-) unit differences among study quarters.

#### Restraint for Fall Prevention

In the second analysis we modeled the odds of a unit reporting restraint use for fall prevention in a quarter. To allow each unit to serve as its own control, this analysis was limited to units having at least one quarter in which restraint use for fall prevention was reported and one quarter in which it was not. This analysis was identical to the first except for the outcome variable and sample.

## RESULTS

Of 39,322 total quarters in the sample data set, 891 (2.3 %) were missing staffing data and 1598 (4.1 %) were missing restraint data. Hospitals enter and leave the NDNQI over time, and units start and may stop reporting restraint data at different times; thus, only quarters with missing restraint data between a unit’s first quarter and last quarter of restraint data reporting during the study period were counted as missing restraint data. Mean age and proportion male were missing for 624 (1.7 %) and 583 (1.5 %) of the 37,724 quarters in which restraint data were reported. Excluding quarters with missing restraint, staffing, mean age, or proportion male data left 36,202 (92.1 %) quarters available for statistical modeling.

There were 3101 units from 869 hospitals in the study sample. Among the 853 hospitals for which demographic data were available, 790 (93 %) were located in metropolitans areas, 411 (48 %) were teaching facilities, and 523 (61 %) had fewer than 300 beds. The Northeast, Midwest, South, and West census regions accounted for 212 (25 %), 99 (12 %), 415 (49 %), and 127 (14 %) hospitals, respectively.

Descriptive statistics for the study sample, the set of units reporting zero restraint use, and the set of units reporting restraint use in every quarter are provided in Table 1. Compared to units in the study sample, units never reporting restraint use tended to be smaller, a larger percentage were surgical units, and a smaller percentage were medical units. Units in the study sample reported 15,014 patients restrained (1.6 %) out of 923,556 patients assessed. Fall prevention was reported as a justification for 7604 (51 %) of these restraints. The average number of patients assessed for restraint per quarterly unit survey was 24.5.

**Table 1:** Descriptive Statistics for Study Sample, Units Reporting no Restraint Use, and Units Using Restraint Each Quarter

	Study sample	Units reporting no restraint use	Units using restraint each quarter
Units	3101	2276	63
Medical	1130 (36 %)	637 (28 %)	22 (35 %)
Surgical	622 (20 %)	671 (29 %)	5 (8 %)
Medical-surgical	1349 (44 %)	968 (43 %)	36 (57 %)
Unit census	25.8 ± 9.7	20.5 ± 8.8	27.9 ± 9.7
Patient age	62.1 ± 7.6	60.5 ± 9.5	62.1 ± 9.6
Percent male	45.8 ± 10.9	42.8 ± 14.6	51.4 ± 15.3
TNHPPD	8.5 ± 1.4	8.9 ± 1.8	8.5 ± 1.7
Skill mix	65.3 ± 9.4	66.4 ± 10.6	66.3 ± 10.6
Prevalence, any reason	1.8 ± 1.9	0	8.5 ± 4.9
Prevalence, fall prevention	0.9 ± 1.4	0	4.0 ± 3.5

Mean ± SD computed across units

Descriptive statistics by staffing category for unit quarters in the two analysis samples are provided in Table 2. There were 1120 (816) medical units, 620 (409) surgical, and 1338 (941) medical-surgical units represented in the first (second) analysis. Restraint rates were highest in quarters for which staffing level or skill mix was below unit average. There was little evidence of association between staffing level and skill mix; correlations between quarterly TNHPPD and skill mix were negligible (r<0.04 in both samples).

**Table 2:** Descriptive Statistics for Quarters in Analysis Samples

	Any restraint			Fall prevention restraint		
	TNHPPD^a^	Skill mix^b^	Rate^c^ (%)	TNHPPD	Skill mix	Rate^d^ (%)
Unit type						
Medical	8.5 ± 1.5	65.1 ± 9.7	1.9 ± 4.7	8.4 ± 1.5	64.8 ± 9.5	1.2 ± 3.4
Surgical	8.6 ± 1.4	65.8 ± 9.1	1.4 ± 4.2	8.6 ± 1.4	65.7 ± 9.1	1.0 ± 3.8
Medical-surgical	8.5 ± 1.6	65.6 ± 10.3	1.8 ± 4.7	8.5 ± 1.6	65.5 ± 10.3	1.3 ± 4.0
TNHPPD category^e^						
Very low (z < −1.25)	7.5 ± 1.3	65.8 ± 10.9	2.1 ± 6.0	7.5 ± 1.3	65.4 ± 10.8	1.5 ± 5.0
Low (−1.25 < z < −0.5)	8.0 ± 1.3	65.9 ± 10.1	1.8 ± 4.9	8.0 ± 1.3	65.7 ± 10.1	1.3 ± 4.6
Average (−0.5 < z < 0.5)	8.5 ± 1.4	65.4 ± 9.5	1.7 ± 4.2	8.5 ± 1.4	65.2 ± 9.5	1.1 ± 3.2
High (0.5 < z < 1.25)	9.0 ± 1.5	65.3 ± 9.5	1.7 ± 4.3	9.0 ± 1.5	65.1 ± 9.4	1.1 ± 3.2
Very high (1.25 < z)	9.6 ± 1.8	64.6 ± 9.7	1.7 ± 4.2	9.6 ± 1.7	64.5 ± 9.7	1.1 ± 3.2
Skill mix category^e^						
Very low (z < −1.25)	8.6 ± 1.6	60.6 ± 9.9	2.2 ± 6.6	8.6 ± 1.6	60.3 ± 9.8	1.5 ± 4.6
Low (−1.25 < z < −0.5)	8.5 ± 1.5	62.3 ± 9.6	2.0 ± 5.1	8.5 ± 1.5	62.1 ± 9.5	1.4 ± 4.3
Average (−0.5 < z < 0.5)	8.5 ± 1.5	65.4 ± 9.1	1.6 ± 3.9	8.5 ± 1.5	65.2 ± 9.1	1.1 ± 3.4
High (0.5 < z < 1.25)	8.5 ± 1.5	68.6 ± 9.1	1.6 ± 4.0	8.4 ± 1.5	68.4 ± 9.0	1.1 ± 3.4
Very high (1.25 < z)	8.4 ± 1.6	70.5 ± 9.6	1.6 ± 4.3	8.4 ± 1.6	70.2 ± 9.7	1.1 ± 3.5
Year						
2006	8.3 ± 1.6	62.6 ± 9.9	2.9 ± 7.4	8.3 ± 1.5	62.5 ± 9.7	3.0 ± 5.4
2007	8.3 ± 1.5	63.5 ± 9.8	2.4 ± 6.3	8.3 ± 1.5	63.3 ± 9.6	2.8 ± 6.6
2008	8.5 ± 1.6	64.4 ± 9.9	1.8 ± 4.2	8.4 ± 1.6	64.0 ± 9.8	2.1 ± 4.6
2009	8.6 ± 1.5	66.2 ± 9.9	1.6 ± 4.3	8.6 ± 1.5	66.0 ± 9.9	1.9 ± 4.8
2010	8.6 ± 1.4	67.2 ± 9.2	1.4 ± 3.4	8.6 ± 1.4	67.2 ± 9.3	1.6 ± 3.4
Quarter						
1	8.3 ± 1.5	65.5 ± 9.8	1.9 ± 4.4	8.3 ± 1.5	65.3 ± 9.7	2.2 ± 4.9
2	8.5 ± 1.5	65.2 ± 9.8	1.7 ± 4.8	8.5 ± 1.5	65.0 ± 9.8	2.0 ± 5.1
3	8.6 ± 1.5	65.4 ± 9.8	1.7 ± 5.0	8.6 ± 1.5	65.2 ± 9.8	2.1 ± 5.5
4	8.6 ± 1.5	65.7 ± 9.8	1.7 ± 4.2	8.6 ± 1.5	65.5 ± 9.8	2.0 ± 4.1

Mean ± SD computed across unit-quarters

^a^TNHPPD = total nursing hours per patient day

^b^Skill mix = % of nursing care hours provided by registered nurses

^c^Rate = % of patients restrained, any justification

^d^Rate = % of patients restrained for fall prevention

^e^Category defined by standardized distance from unit mean: very low (>1.5 SDs below), low (0.5–1.25 SDs below), average (within 0.5 SDs), high (0.5–1.25 SDs above), and very high (>1.5 SDs above)

We found statistically significant effects of skill mix category on odds of any restraint (*p* < 0.001) and odds of fall prevention restraint (*p* = 0.035). Compared to quarters with average skill mix, adjusted odds of any restraint use were 11 % and 18 % higher, respectively, for quarters with low and very low skill mix (see Table 3). Odds of fall prevention restraint were 9 % and 16 % higher, respectively, for quarters with low and very low skill mix. Mean skill mix was also a significant predictor of both types of restraint; a unit with 10 percentage points higher skill mix than an otherwise equivalent unit was estimated to have 13 % lower odds of any restraint and 7 % lower odds of fall prevention restraint.

**Table 3:** Results of Full Logistic Regression Models for Odds of Restraint Use

Variable	Level	Any restraint	Fall prevention restraint
		Adjusted OR (95 % limits)^a^	Adjusted OR (95 % limits)^b^
TNHPPD category	Very low	1.06 (0.96–1.16)	1.12 (1.00–1.26)
	Low	1.07 (1.00–1.14)	1.09 (1.00–1.19)
	Average	Referent	Referent
	High	1.06 (0.98–1.13)	1.02 (0.93–1.11)
	Very high	0.99 (0.91–1.09)	1.05 (0.93–1.18)
Skill mix category	Very low	1.18 (1.08–1.29)	1.16 (1.03–1.29)
	Low	1.11 (1.04–1.19)	1.09 (1.00–1.19)
	Average	Referent	Referent
	High	1.03 (0.96–1.10)	1.00 (0.91–1.09)
	Very high	0.98 (0.89–1.08)	0.98 (0.86–1.10)
Mean TNHPPD		1.02 (0.98–1.05)	0.97 (0.93–1.01)
Mean skill mix		0.87 (0.82–0.92)	0.93 (0.88–0.98)
Patient days		1.33 (1.26–1.39)	1.24 (1.17–1.31)
Average age		1.02 (1.02–1.03)	1.02 (1.01–1.02)
Proportion male		1.65 (1.34–2.02)	1.44 (1.12–1.85)
Unit type	Medical- surgical	1.35 (1.22–1.49)	1.25 (1.12–1.41)
	Medical	1.31 (1.19–1.44)	1.16 (1.03–1.29)
	Surgical	Referent	Referent
Bed size	≥300 beds	1.11 (0.99–1.26)	1.08 (0.96–1.22)
Location	Metropolitan	1.37 (1.07–1.74)	1.18 (0.90–1.54)
Teaching status	Teaching	1.22 (1.08–1.38)	1.12 (0.99–1.26)
Census region	West	1.46 (1.17–1.82)	1.37 (1.09–1.73)
	South	1.27 (1.06–1.52)	1.20 (0.99–1.44)
	Midwest	1.05 (0.86–1.28)	1.02 (0.83–1.26)
	Northeast	Referent	Referent
Year	2006	1.92 (1.62–2.27)	1.87 (1.52–2.30)
	2007	1.59 (1.47–1.73)	1.64 (1.49–1.82)
	2008	1.25 (1.16–1.35)	1.31 (1.19–1.44)
	2009	1.07 (1.00–1.15)	1.16 (1.06–1.27)
	2010	Referent	Referent
Quarter	1	1.17 (1.09–1.26)	1.15 (1.05–1.26)
	2	1.02 (0.95–1.10)	0.99 (0.90–1.08)
	3	1.02 (0.95–1.09)	1.02 (0.93–1.12)
	4	Referent	Referent

^a^C-statistic = 0.77

^b^C-statistic = 0.73

To examine how the effects of skill mix category were influenced by the presence of various explanatory variables, we fit the following models for comparison: a base model (including year, quarter, patient volume, mean skill mix, and skill mix category as explanatory variables), base model plus hospital variables (census region, bed size, location, teaching status), base model plus unit variables (unit type, mean TNHPPD, TNHPPD category), base model plus patient mix variables (mean age, proportion male), and a full model including all explanatory variables. Odds ratios for skill mix category changed very little across models (see Table 4).

**Table 4:** Adjusted Odds Ratios (95 % Limits) for Skill Mix Categories Across Comparison Models

Skill mix category	Base	Base + Hospital	Base + Unit	Base + Patient	Base + All
	Any restraint				
Very low	1.17 (1.07–1.28)	1.17 (1.07–1.28)	1.17 (1.07–1.28)	1.18 (1.08–1.29)	1.18 (1.08–1.29)
Low	1.11 (1.04–1.19)	1.11 (1.04–1.19)	1.11 (1.04–1.19)	1.11 (1.04–1.19)	1.11 (1.04–1.19)
Average	Referent	Referent	Referent	Referent	Referent
High	1.03 (0.96–1.11)	1.03 (0.96–1.10)	1.03 (0.96–1.10)	1.03 (0.97–1.11)	1.03 (0.96–1.10)
Very high	0.99 (0.91–1.09)	0.99 (0.90–1.09)	0.99 (0.90–1.09)	0.99 (0.90–1.09)	0.98 (0.89–1.08)
	Fall prevention restraint				
Very low	1.17 (1.04–1.30)	1.16 (1.03–1.30)	1.16 (1.04–1.30)	1.17 (1.04–1.30)	1.16 (1.03–1.29)
Low	1.09 (1.00–1.18)	1.09 (1.00–1.18)	1.09 (1.00–1.18)	1.09 (1.00–1.18)	1.09 (1.00–1.19)
Average	Referent	Referent	Referent	Referent	Referent
High	1.01 (0.92–1.10)	1.00 (0.92–1.10)	1.00 (0.92–1.09)	1.01 (0.92–1.10)	1.00 (0.91–1.09)
Very high	0.98 (0.87–1.11)	0.99 (0.88–1.12)	0.97 (0.86–1.10)	0.98 (0.87–1.11)	0.98 (0.86–1.10)

Although adjusted odds of fall prevention restraint were 9–12 % higher in quarters with below-average staffing levels, staffing level category was not a statistically significant predictor of fall prevention restraint (*p* = 0.160) or of any restraint (*p* = 0.250). Associations between mean TNHPPD and odds of restraint use were also non-significant.

Study year was a statistically significant predictor of fall prevention restraint and any restraint (*p*s < 0.001). As shown in Table 3, odds of both types of restraint decreased each year, as did restraint prevalence rates, which fell by about 50 % during the study period (see Table 2). Quarter also predicted use of fall prevention (*p* = 0.004) and any restraint (*p* < 0.001). This effect was driven by higher odds of restraint in the first quarter (see Table 3).

## DISCUSSION

Using longitudinal data from over 3000 units, we found that odds of physical restraint use were lower for units with higher mean skill mix. Further, restraint odds were higher when a unit’s quarterly skill mix was below its average level across study quarters, but not significantly lower when a unit’s skill mix was high or very high relative to average. These findings held for both fall prevention restraints and restraints in general.

Consistent with one previous study,^28^ our findings suggest that skill mix, not total staffing level, is the more important predictor of restraint use and that patient care quality may suffer when unit staffing models cannot respond to changes in patient volume or RN availability except by increasing non-RN hours. This is further evidence that the type of nursing staff, not just the number of staff per patient, can be important for patient outcomes.^21^^,^ 22 ^,^ 31 ^–^ 33 Nurses must obtain a physician order for restraint, and having an adequate proportion of RNs apparently reduces the likelihood of nursing staff requesting such an order, perhaps because RNs are better trained to find alternatives to restraint. In any case, restraint involves both nurses and physicians, and reduction in restraint use must be a collaborative effort.

We found little evidence that higher total staffing levels are associated with lower odds of restraint use. In California, the impact of mandated minimum nurse-to-patient ratios on quality has been mixed,^34^^–^ 36 and studies of the effect of staffing levels on restraint use have yielded inconclusive results.^29^^,^ 30 ^,^ 37

One possible explanation for mixed results in studies of staffing level and restraint use is lack of control for unmeasured differences among units. Perhaps some units have both higher staffing levels and higher restraint rates simply because they have sicker patients, resulting in a spurious, positive correlation between staffing and restraints. Whatever the explanation, the difficulty of establishing an adequate risk adjustment model for a complex phenomenon such as restraint use highlights the advantage of our approach, in which units serve as their own control.

Restraint prevalence and odds of restraint decreased substantially during the study period, suggesting that efforts to reduce restraint use have met with some success. Whether this encouraging trend has continued since 2010 is a question for further study. We also observed a seasonal trend, with elevated prevalence and odds of restraint in the first quarter. Rates of hospital-acquired pressure ulcers are also highest in the first quarter,^38^ suggesting patients tend to be sicker during these winter months.

### Limitations

One limitation of our study is the non-random sample, which may not be representative. Larger hospitals and Magnet-designated facilities are disproportionately represented in the NDNQI, for example. However, we controlled for several hospital characteristic variables in our study, and we doubt that staffing-restraint associations differ enough across hospital sizes and types for the non-random nature of our sample to undermine the generalizability of our basic findings.

Our quarterly data had limited granularity. The size of the effects we observed despite this limitation suggests a robust association between skill mix and restraint use. We would expect analyses of week- or shift-level data to yield similar results, but the NDNQI does not collect data at this level, so this is a question for further research.

Another study limitation was the lack of data on potentially important covariates, including patient acuity and fall risk, units’ use of non-nursing staff such as sitters to prevent falls, and the presence of family in the patient’s room. We could not fully control for between- or within-unit differences on these covariates, but our use of each unit as its own control makes this less of a concern. Moreover, the effect of skill mix category was remarkably constant regardless of the covariates included in the model, despite some of these covariates having substantial effects in their own right (see Table 4).

Our approach of using each unit as its own control required each unit analyzed to have at least one quarter with and one quarter without restraint use, limiting our sample of units. However, our study sample was comparable in most respects to the set of units excluded for never reporting restraint use, and we think the staffing-restraint use association we found likely holds quite generally among units where restraints are used at least on occasion. In future research, examination of data from units reporting restraint use in every quarter would likely yield further insight into the relation between staffing and restraint use.

### Strengths

In addition to the very large sample size, our methodological approach is an important strength of this study. Nurse staffing research typically involves cross-sectional comparisons of staffing levels across units, which tend to reflect between-unit differences in patient mix (e.g., average risk of adverse outcome). By contrast we examined the effects of within-unit differences in quarterly staffing, effectively using each unit as its own control, by computing each unit’s quarterly nurse staffing level and skill mix based on standard deviations from the unit’s average, thereby allowing for comparisons that implicitly take into account unmeasured unit characteristics. This approach alleviates the confounding that occurs when effects due to between-unit differences in patient mix are attributed to between-unit differences in staffing. Of course, patient mix can change from quarter to quarter on a given unit, but this within-unit variability is arguably less of a confounding concern than the variability among units. This approach allowed us to move beyond comparisons of *units* with higher versus lower staffing levels or skill mix to identify *quarters* with below-average skill mix as having higher rates of restraint use.

## Conclusion

We found that when unit skill mix was low, hospital staff were apparently more likely to use restraints, including restraints for fall prevention. Above-average skill mix, however, was not associated with reduced restraint use. The key seems to be ensuring that the skill mix is consistently adequate.

US hospitals have little buffer for surges in demand for nurse staffing.^39^ In light of a projected RN shortage, future research on nurse-sensitive outcomes, including restraint use, should focus on optimizing available nursing hours at the bedside through innovative staffing models and improvements in the work environment.^40^^,^ 41
